# The early evolution of land plants, from fossils to genomics: a commentary on Lang (1937) ‘On the plant-remains from the Downtonian of England and Wales'

**DOI:** 10.1098/rstb.2014.0343

**Published:** 2015-04-19

**Authors:** Dianne Edwards, Paul Kenrick

**Affiliations:** 1 School of Earth and Ocean Sciences, Cardiff University, Cardiff CF10 3AT, UK; 2 Department of Earth Sciences, The Natural History Museum, Cromwell Road, London SW7 5BD, UK

**Keywords:** tracheophyte, bryophyte, *Cooksonia*, cryptogamic cover, Siluro-Devonian, terrestrialization

## Abstract

During the 1920s, the botanist W. H. Lang set out to collect and investigate some very unpromising fossils of uncertain affinity, which predated the known geological record of life on land. His discoveries led to a landmark publication in 1937, ‘On the plant-remains from the Downtonian of England and Wales’, in which he revealed a diversity of small fossil organisms of great simplicity that shed light on the nature of the earliest known land plants. These and subsequent discoveries have taken on new relevance as botanists seek to understand the plant genome and the early evolution of fundamental organ systems. Also, our developing knowledge of the composition of early land-based ecosystems and the interactions among their various components is contributing to our understanding of how life on land affects key Earth Systems (e.g. carbon cycle). The emerging paradigm is one of early life on land dominated by microbes, small bryophyte-like organisms and lichens. Collectively called cryptogamic covers, these are comparable with those that dominate certain ecosystems today. This commentary was written to celebrate the 350th anniversary of the journal *Philosophical Transactions of the Royal Society*.

## 1. Introduction

Plants are of fundamental importance to life on land, so developing an understanding of their origins and early evolution has been a quest for generations of botanists. Once the domain of morphology and palaeontology, recent progress in comparative genomics and molecular developmental biology brings new tools to our understanding of relationships among species and the development of their organs and tissues. At the same time, a growing understanding of the composition of early terrestrial ecosystems and the interactions among various components (e.g. plants, fungi, arthropods) is shedding light on how the evolution of life on land affected key Earth Systems (e.g. carbon cycle). Advances in these areas are driving a resurgence of interest in the earliest fossilized remains of land living organisms, whose study was initiated by W. H. Lang and published in 1937 (On the plant-remains of the Downtonian of England and Wales) [1].

**Figure 1.**
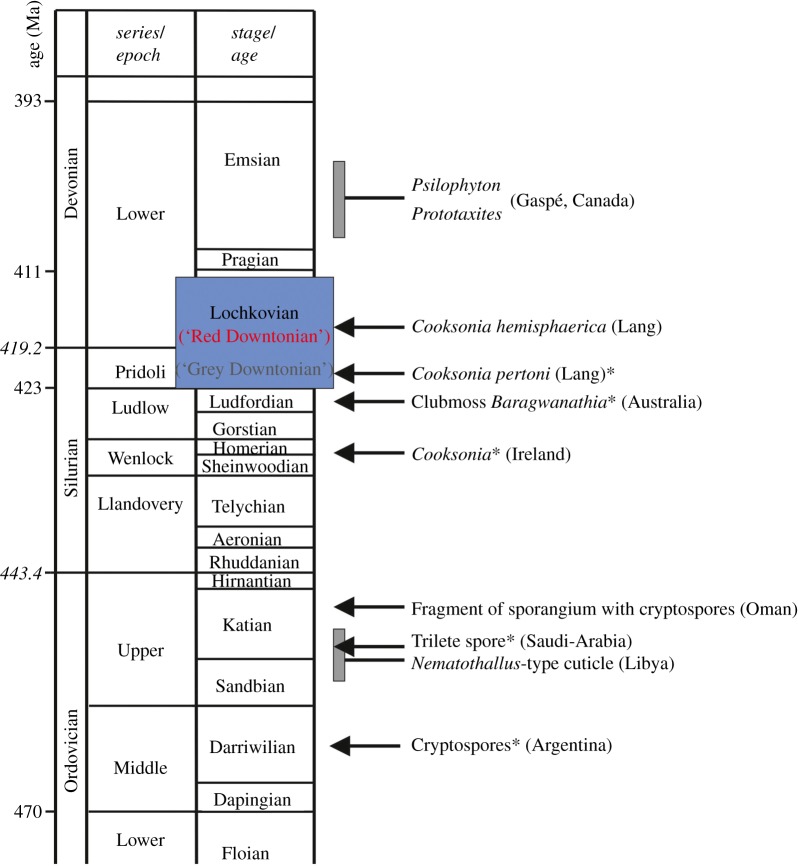
Stratigraphic occurrences of fossil plants mentioned in text. Ages in millions of years are taken from the *International Chronographic Chart of the International Commission on Stratigraphy*, 2012. All fossils mentioned are earliest occurrences (*), except for *Psilophyton* and *Prototaxites*, which indicate the age of the first specimens described by Dawson. Coloured block represents the time interval in Lang's paper: note that his ‘Downtonian′ encompasses the Pridoli Series (Grey Downtonian) and most of the Lochkovian Stage (Red Downtonian).

Knowledge of modern organisms is essential to interpreting fossils, but preconceptions can also be a hindrance. Lang's paper followed pioneering earlier works on slightly younger rock sequences from Europe and Canada (figure 1) [2,3]. Here the fossil record revealed that prior to the origins of forest ecosystems early plants differed in notable ways from those of later floras and especially from modern species. For example, many lacked such characteristic organs as leaves and roots, making them difficult to recognize as plants and as a consequence potentially attributable to a wide range of other groups, including marine algae. They were also small—generally less than a metre in height—and their simple bifurcating stems bore novel spore-producing organs. Questions regarding the land living status of these fossils were answered beyond doubt with the discovery of specimens exquisitely preserved in silicates in the Rhynie Chert (Scotland) [4]. The presence of such structures as epidermis with stomata and a primitive vascular system together with the environmental context confirmed that despite the complete absence of certain categories of organ these were plants that lived on land. Drawing on this emerging body of new data and interpretation, Lang took these investigations significantly further. Geologists were unearthing organic remains in still older rocks, and these mostly defied classification in any known group of organisms. They were very challenging to interpret, because they were very small, highly fragmentary, and they varied greatly in shape and texture. Yet, they were highly abundant in certain types of sediment. Lang set out to collect and investigate this unpromising material, employing microscopy and techniques that had recently been developed to extract organics from sedimentary rocks. Cast to one side by previous generations of geologists, Lang showed that these apparently nondescript organic remains were a goldmine of data on early land ecosystems.

One of its referees, A. C. Seward FRS, the leading palaeobotanist of his generation, wrote ‘This paper is exceptional in its botanical and geological importance; in it are described the remains of the oldest known British flora and considering the unpromising nature of the material, the information obtained is amazing.′ Indeed, it was a model of its time in that in addition to exhaustive anatomical investigations of the plant fossils, he meticulously recorded the geological strata in which they were preserved and, using the nature of the rock record combined with information from animal fossils, he reconstructed depositional environments and established that the plants together with less easily categorized fossils derived from the land. It should be emphasized that all of the fossils had been transported and buried in either shallow, near shore marine sequences (Upper Silurian) or terrestrial sediments deposited by river systems (Lower Devonian) [5].

## 2. Lang's achievements

### (a) Early vascular plants

**Figure 2.**
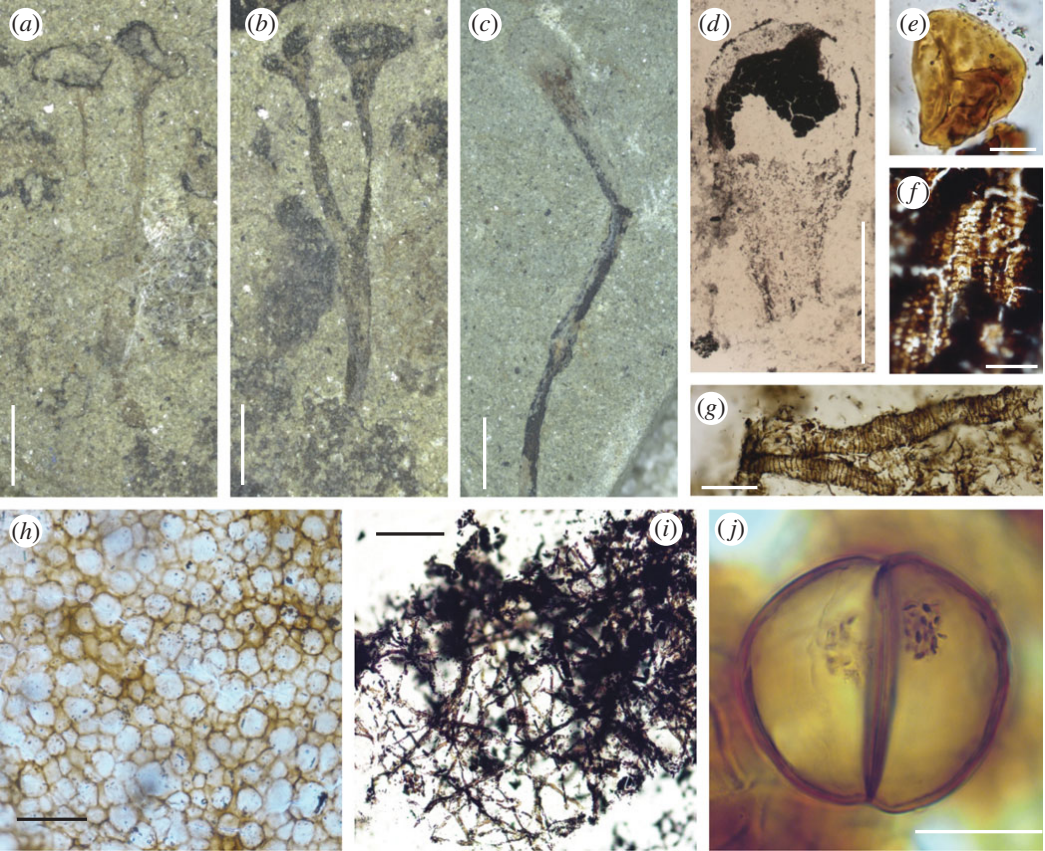
New light micrographs of material illustrated by Lang [1]. Original number and figure numbers in brackets. (*a*) *Cooksonia pertoni.* Lectotype. Přídolí. V58011. (124 Z b; Plate 8, fig. 8). Coalified material has been removed, possibly using cellulose acetate, for further analysis. Scale bar, 2 mm. (*b*) Counterpart of (*a*). V58010. Black patches possibly of *Nematothallus*. Scale bar, 2 mm. (*c*) *Cooksonia hemisphaerica*. Lochkovian. V58022. (186; Plate 9, fig. 33). Scale bar, 2 mm. (*d*) *C. hemisphaerica* sporangium recovered using cellulose acetate. V54592. (1062; Plate 9, fig. 34). Scale bar, 1 mm. (*e*) Spore recovered from a sporangium of *C. pertoni*. V54995. (1105: Plate 8, fig. 11). Scale bar, 10 μm. (*f*) Tracheids recovered using cellulose acetate from a sterile axis, thought by Lang to belong to *C. hemisphaerica.* Lochkovian. V55040. (1150; Plate 9, fig. 36). Scale bar, 50 μm. (*g*) Banded tubes on cellulose acetate sheet. Přídolí. (962; Plate 11, fig. 60). Scale bar, 50 μm. (*h*) *Nematothallus* ‘cuticle′ on a cellulose acetate sheet. Přídolí. V54697. Scale bar, 50 μm. (*i*) Typical wefts of tubes assigned to *Nematothallus*. V54851. (Not figured, but cf. plate 11). Scale bar, 160 μm. (*j*) Dyad in elongate cylindrical spore mass. Přídolí. V54654. (764; Plate 13, fig. 109). Granular material probably represents condensed cell contents. Scale bar, 20 μm.

The most enduring of Lang's achievements was his discovery and characterization of the plant he named *Cooksonia*. Fossils attributable to this genus have slender bifurcating systems, measuring from about 1 cm to no more than 6 cm in length (figure 2*a–c*), that completely lack leaves of any kind. Each is composed of several parallel-sided axes that terminate in distinctive ellipsoidal structures (figure 2*b*). Remarkably, Lang was able to prove that these were spore-bearing organs (sporangia) by extracting preserved spores, which measured between 25 µm and 38 µm in diameter. Technically difficult at the time, this was achieved with the application of transparent cellulose acetate, to which the spores adhered, enabling them to be removed from the sporangia and examined using light microscopy. Lang showed that the spores were smooth-walled, but that each bore a distinctive trilete (Y) mark (figure 2*e*), indicating that they were produced by meiosis. Based on the evidence of these fragmentary remains Lang named his new plant *Cooksonia pertoni* and concluded that it was undoubtedly land dwelling. Further clarification of its affinities required crucial evidence of the plant's tissue systems, but because the fossils were preserved as thin coaly films, little of the original cellular structure remained. Several of the earliest sites (Late Silurian Period) yielded associated fragments of axis that showed the faint outlines of elongate cortical cells coupled with a distinctive central strand of thicker carbonaceous material hinting at the presence of vascular tissues. A second species, *C. hemisphaerica* (figure 2*c,d*), was described from a slightly younger site (Early Devonian Period). Here, Lang found numerous axial fragments which he considered ‘with practical certainty′ belong to *C. hemisphaerica*. One such possessed a central strand composed of the degraded remains of what he interpreted were tracheids with annular thickenings (figure 2*f*). Lang pronounced this crucial evidence as ‘the most ancient piece of vascular tissue as yet demonstrated in position in a fossil plant in Britain′. Thus, by inference, he concluded that both species of *Cooksonia* were related to the vascular plants, a group that today includes among others ferns, conifers and flowering plants. Lang's discovery and meticulous observations revealed a diversity of small fossil plants of great simplicity. *Cooksonia* has since become an iconic fossil thought to demonstrate the archetypal body plan of primitive vascular plants.

### (b) Spores

In addition to the individual spores that Lang recovered from the sporangia of *Cooksonia*, he also discovered within the same sediments isolated clusters or masses of spores of varying shapes and sizes. Most clusters were circular or oval, measuring 0.5–1.2 mm. One elongate specimen, measuring 4.5 mm in length and approximately 1 mm in width, contained hundreds of spores. Lang surmised that these were also derived from land plants, but that the enclosing tissue systems had decomposed whereas the spores, being more robust, had survived. The shapes of the spore masses thus reflected the shapes of the original plant's spore-bearing organs. Intriguingly, one elongate mass contained highly unusual spores each with two hemispherical cells separated by a transverse wall, which Lang described as bicellular (figure 2*j*). This contrasts with the normal condition in land plants, in which spores develop in tetrads following meiotic cell division. Lang considered this ‘unique among pre-Carboniferous spores with which I am acquainted′ and entertained the possibility that they demonstrated the first stage of germination. These spores are now known to be dispersed as permanently fused dyads, and they are an abundant element of the microscopic organics extractable from sediments of the Late Ordovician through to the Early Devonian.

### (c) Nematothallus

Arguably, the most challenging and controversial of Lang's discoveries was the organism that he named *Nematothallus pseudovasculosa*, which was abundant at many of his sites taking the form of dark ‘incrustations' (figure 2*a,b*). Lang showed that these were composed of wefts of tubes covered by a resilient film of ‘cuticle′ with a pseudocellular patterning (figure 2*h*). The tubes were highly distinctive and of two size classes: narrow tubes (*ca* 2.5 µm) and wide tubes (12–40 µm; figure 2*i*). They bifurcated occasionally. On the internal wall of the wider tubes there was evidence of narrow annular bands (figure 2*g*). Reconstructing the three-dimensional organization in such fossils is difficult, but from consistent associations, too frequent to be coincidental, Lang considered the ‘plant′ to be thalloid with the wefts of fine tubes sandwiched between the uppermost pseudocellular cuticle and more deeply seated wefts of larger tubes. On decay these layers would have separated, thus accounting for their frequent recovery as isolated elements from the rock matrix. Lang also deduced that *Nematothallus* reproduced by means of spores, again based on the consistent association of tubes and spores of variable shape and surface ornament. Having worked out the general structure of *Nematothallus*, Lang was then faced with the challenge of understanding the affinities of an organism whose tubular structure defied comparison with plants, or as he put it: ‘makes comparison with all plants of typical cellular structure open to serious criticism′.

Among the remains collected by Lang, there was another obscure but highly distinctive fossil that had earlier been described by Dawson [2] based on Canadian material, and which was widely encountered in Late Silurian and Early Devonian sediments. Named *Prototaxites*, it consisted of axes that ranged enormously in size from a few millimetres to a staggering 1 m in diameter and 8 m in length [6]. Like *Nematothallus*, it was constructed from profusely branched tubes of various sizes. The affinities of *Prototaxites* were vigorously debated, but starting with Carruthers [7] there was a growing consensus that its affinities lay with the algae, and because of its great size and tissue complexity, probably with the brown algae. An alternative view, overlooked by Lang, was the suggestion by Church [8] that *Prototaxites* was a giant fungus, a hypothesis recently resurrected by Hueber [6]. Because of similarities in their underlying structure (i.e. tissues comprising profusely branched tubes) and their frequent close association in sediments, Lang explored the possibility of a physical relationship between the two—could *Nematothallus* be the laminate appendages of the ‘trunks' of *Prototaxites*? He decided to unite the two genera in a new ‘class', the Nematophytales. Lang was convinced that *Nematothallus* lived on land as evidenced by its spores and cuticle. Having reviewed most of the published opinions, he concluded that nematophytes composed a unique grouping of land plants being neither algae nor vascular plants, although in possession of some characteristics of both groups. Despite major uncertainties surrounding its affinities and its functional biology, Lang himself thought of his work on *Nematothallus* as being ‘most important to our botanical knowledge’.

## 3. Advances

### (a) *Cooksonia* today

**Figure 3.**
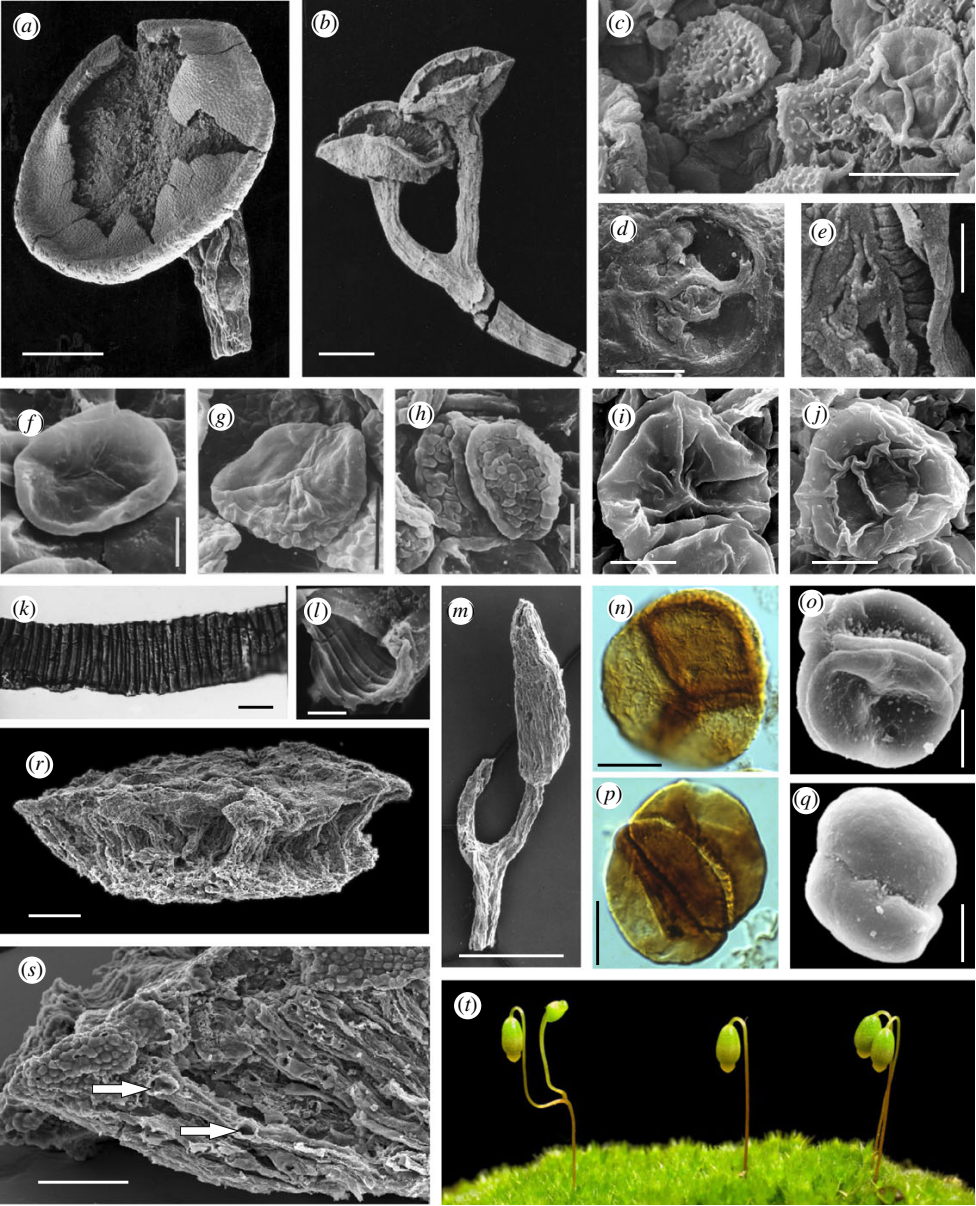
All scanning electron micrographs except where stated. (*a*) *Cooksonia pertoni* subsp. *apiculispora*. Lochkovian. North Brown Clee Hill, Shropshire. Scale bar, 500 µm. First published in [10, fig. 1a]. Museum number NMW94.60G.17. (*b*) *C. pertoni* subsp. *apiculispora* Lochkovian. North Brown Clee Hill, Shropshire. Scale bar, 500 µm. First published in [12, plate III-1]. Museum number NMW94.60G.14. (*c*) Proximal (extreme right) and distal surfaces of spores of *Aneurospora newportensis*, isolated from sporangium in (*a*). Scale bar, 20 µm. From [10, fig. 1d]. (*d*) Stoma from subtending stem in (*a*). Scale bar, 20 µm. From [10, fig. 1b]. (*e*) Cast of tracheid from stem in (a); grooves indicate position of annular thickenings. From [10, fig. 2a]. (*f*)–(*j*) Spores present in subspecies of *C. pertoni*. (*f*) Proximal surface of *Ambitisporites* sp. (subsp. *pertoni*), Upper Silurian. Scale bar, 10 µm. First published in [13, fig. 3.1b]. V.62776. (*g,h*) Proximal and distal surfaces of *Synorisporites verrucatus* (subsp. *synorispora*), Upper Silurian. Scale bar, 10 µm. First published in [14; plate 1, figs 3, 4]. NMW93.143G.1. (*i,j*) Proximal and distal surfaces of *Synorisporites* sp. (subsp. *reticulispora*), Lochkovian. Scale bar, 10 µm. First published in [15, plate VI, 3, 18]. NMW2012.29G.20 and 19. (*k*) Light micrograph of a tubular structure (?hypha/banded tube) with internal spiral thickenings isolated from late Wenlock (Silurian), Rumney, Cardiff. Scale bar, 10 μm. First published in [16, fig. 23]. MPK 6028. (*l*) Fractured end of banded tube, Ludlow (Upper Silurian), S. Wales. Scale bar, 5 µm. First published in [17, fig. 68]. NMW77.34G.33c. (*m*) *Fusitheca fanningiae* containing permanent laevigate dyads with thin envelope. Lochkovian, Shropshire. Scale bar, 500 µm. First published in [18, fig. 54]. NMW97.42G.4. (*n*) Light micrograph of *Velatitetras rugulata*, a permanent tetrad enclosed in a ‘rucked’ envelope, isolated from Wenlock rock, Shropshire. Unpublished–courtesy of Neil Burgess. Scale bar, 10 μm. (*o*) *Tetrahedraletes medinensis*, a permanent tetrad, Ordovician, Shropshire. Scale bar, 13 µm. First published in [19, fig. 5A]. (*p*) Light micrograph of *Artemopyra brevicosta*, a permanent dyad, Wenlock, Shropshire. Scale bar, 10 µm. First published in [20; plate 1, fig. 1]. (*q*) *Dyadospora murusdensa*, a permanent dyad, Ordovician, Shropshire. Scale bar, 10 µm. First published in [19; plate 2, fig. 11]. (*r*,*s*) Fragment of *Nematothallus williamii*, Lochkovian, Shropshire. First published in [21, fig. 1A,B]. Museum number NMW2013.39G.1. (*r*) Note three-layered thallus. Scale bar, 200 µm. (*s*) Magnification of (*r*) showing the surface patterning typical of the *Nematothallus* ‘cuticle’ and larger hyphae aligned perpendicular to the surface. Arrows indicate positions of lateral branches or areas in contact with a postulated photobiont. Scale bar, 100 µm. (*t*) Gametophytes and fertile sporophytes of *Funaria hygrometrica.* Note bifurcating seta in sporophyte on extreme left. (Courtesy of Jill Harrison, Yoan Coudart and Alison Reed, Cambridge University.)

Since the original discovery by Lang in 1937 research has focused on developing a better understanding of the diversity of plants attributable to *Cooksonia* and in particular the nature of their internal tissue systems to provide a clearer taxonomic concept [9], and an understanding of its functional biology [10] and relationship to living species [11]. Intensive re-collecting at a number of Lang's localities has unearthed a far more diverse assemblage of *Cooksonia*-like plants. Most variation centres on the form of the sporangia and the spores. Preserved stem anatomy is rare, but lingering doubts as to the vascular status of the genus were settled with the discovery of remarkable new fossils of *C. pertoni* preserved in charcoal (figure 3*a,b*). These were recovered from sediments using acid extraction methods. The three-dimensional structure of the plant is faithfully preserved, including cellular level details of tracheids and stomata (figure 3*a–e*) [10]. Furthermore, intensive collecting through an extended stratigraphic interval (i.e. Lang's Downtonian = Upper Silurian (Přídolí) through Lower Devonain (Lochkovian); figure 1) demonstrated that whereas external form is highly conserved, there are recognizable changes at the cellular level. For example, *C. pertoni* produced different kinds of spores in different geological intervals [22]. The spores themselves were structurally similar (i.e. equatorially thickened) but either smooth-walled or differently ornamented (figure 3*f–j*). Such cryptic evolution in plants with simple morphology might also encompass changes in other anatomical features, particularly those relating to the vascular system (e.g. tracheids) and the epidermis (e.g. stomata). Recent research has therefore greatly expanded our knowledge of the morphology and diversity of plants attributable to *Cooksonia*, confirming and extending Lang's original concept so that *C. pertoni* is now one of the best known of the earliest stem-group vascular plants. Nevertheless, frustratingly, newly discovered types preserved in charcoal are highly fragmentary and frequently they are represented only as unique specimens.

### (b) Palaeophysiological approaches

**Figure 4.**
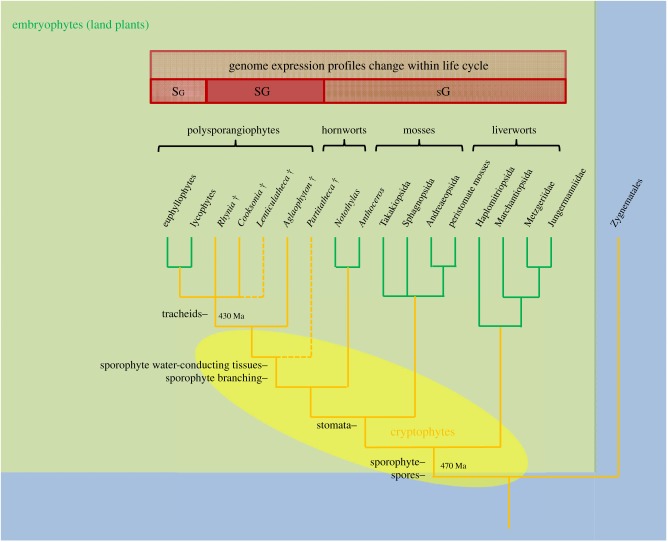
Relationships among major groups of land plants showing the hypothesized broad range of clades to which cryptophytes (extinct cryptospore-producing plants) might belong (shaded yellow oval; modified after [5,26]). As currently envisaged, cryptophytes is a grade of organization potentially encompassing plants spanning the embryophyte to vascular plant stem-groups as well as stem-group hornworts, mosses and liverworts. Some key developments discussed in the text are indicated on branches. Also indicated is a major shift in life cycle among clades from gametophyte dominated (sG) to sporophyte dominated (Sg) life cycles. The intermediate isomorphic life cycle (SG) is only known in some extinct plants. Estimated divergence times in millions of years.

Our growing understanding of the nature of *Cooksonia* has stimulated thinking about the origin and evolution of key land plant characteristics, and how such small plants could have functioned. One area of interest has been the extent to which early plants were capable of maintaining an internally hydrated environment under water stress (homoiohydry) as opposed to having the capacity to tolerate significant desiccation (poikilohydry). Raven addressed this question by taking an ecophysiological approach to characterizing water transport and gas exchange processes in living vascular plants (homoiohydric) and bryophytes (poikilohydric) [23], examining interactions between the demands of photosynthesis and water delivery as plants grew in stature. Results were used to appraise the anatomy and chemical innovations of early land plants starting with *Cooksonia*. Key elements of this analysis included the structure and position of water-conducting cells (tracheids), the synthesis of lignin (wood) and especially the function of stomata, intercellular spaces, and the cuticle system. Raven initially favoured the earlier evolution of internal conducting tissues as a key innovation in the origin of homoiohydry [23,24]. (i.e. tracheids must have evolved before stomata). However, later he saw ecophysiological advantages to ‘stomata first’ in very small sporophytes [25], a conclusion supported by subsequent phylogenetic analyses (see below and figure 4) and the order in which these features appear in the fossil record [27]. The small size of some cooksonias potentially restricts their ability to develop diverse tissues systems with specialized functions. Noting that Raven's analyses had concentrated on adaptations relating to transport processes in axes at the wider end of the range distribution (more than 1 mm diameter), Boyce [28] looked at the nature and functions of tissue systems in narrower axes and concluded that those involved in support (i.e. peripheral strengthening tissues) and water-conduction would have occupied the bulk of the plant body. In smaller species of *Cooksonia*, therefore, there would have been little room for photosynthetic tissues. In addition, *Cooksonia* had few stomata. Thus, Boyce concluded that smaller species were probably not homoiohydric and also not photosynthetically competent. As in modern bryophytes, they must have been physiologically dependent on a gametophyte, but compelling fossil evidence has yet to be found.

The challenge of interpreting the functional biology of *Cooksonia* raises questions about the function and molecular regulation of basic organs and tissue systems in plants. It has been assumed that stomata and cuticle had similar functions and controls in bryophytes, vascular plants and early fossils such as *Cooksonia*. Recently, the role played by the phytohormone abscisic acid (ABA) in stomatal physiology has been questioned, challenging conventional views. The stomata of *Lycopodium* and *Pteridium* were shown to lack a pore closure response to ABA [29]. Similarly, stomata in hornworts and *Sphagnum* do not respond significantly to ABA, desiccation and darkness, and there is no potassium flux between guard cells and epidermal cells [30]. By contrast, in the moss *Physcomitrella* and in the lycopod *Selaginella* stomatal responses to ABA appear to be similar to those in the angiosperm *Arabidopsis* [31,32]. The issue of when active stomatal control in plants evolved is therefore controversial and the subject of active research.

Unlike in the vascular plants, the stomata of some hornworts and mosses are known to open but then never close. Stomata in these plants may therefore have had rather a different function, perhaps aiding the desiccation of the sporangium prior to spore release [30]. In *C. pertoni* stomata might have played a similar role or perhaps they functioned in creating a transpiration stream for mineral nutrition of the sporangium [27]. There is also a functional dependency between stomata and cuticle. Some moss sporophytes share similarities in cuticle ultrastructure with vascular plants, whereas their gametophytes have thinner less well-developed cuticles [33,34]. This is most probably a consequence of the absence of stomata in the gametophyte, meaning that gaseous diffusion must take place through the cuticle, limiting its development. Thus, the function of stomata in early fossil land plants and in living bryophytes is very different to that in modern flowering plants.

Genomics is also beginning to provide interesting insights into the evolution of the stomata, cuticle and vascular systems. It has been shown that a homologue of the ABA regulatory protein kinase (OST1) present in *Physcomitrella* can rescue an *Arabidopsis* mutant lacking OST1 activity, and transcriptome analyses of the moss sporophyte suggest a deeply conserved mechanism of stomatal control [31]. Similarly, knockout mutants of moss ABCG transporter Pp-ABCG7, which has a putative orthologue in *Arabidopsis* involved in cuticle precursor trafficking, are severely deficient in cuticular wax [34]. PpVNS proteins have been shown to regulate the same gene families in *Physcomitrella* and *Arabidopsis*, supporting the hypothesis of a fundamentally similar genetic basis for the development of water-conducting tissues and supporting tissues in both [35]. So, although the physiological roles played by stomata and to a lesser extent cuticle and water-conducting cells are likely to vary across land plants, certain aspects of their development are deeply conserved.

### (c) The phylogeny of early land plants: the bryophyte connection

The curious bicellular spores discovered in spore masses by Lang are now known to belong to a class of organics called cryptospores. This encompasses spores dispersed in permanently fused groups of two (i.e. dyads; figure 3*p,q*) or four (i.e. obligate tetrads; figure 3*n,o*) and their derivatives. Cryptospores are now known to be abundant and cosmopolitan as dispersed forms in rocks of the Ordovician through Devonian Periods [36,37]. The nature and affinities of the plants that produced the cryptospores are still contentious, but knowledge of these is essential to understanding the evolution of early land floras [38]. Progress has come through the comparative analysis of spore morphology and ultrastructure [39], the recovery of spore masses from more ancient rocks [40] and especially through the discovery of cryptospores *in situ* [41]. The leading hypothesis aligns cryptospores with bryophytes. Initially, this was based on parallels between early fossil obligate tetrads and similarly configured spores known to occur in a few modern liverworts (e.g. *Riccia*, *Sphaerocarpos*, *Cryptothallus*). Studies of cell wall ultrastructure (e.g. configuration of wall lamellae) show additional similarities lending support to this hypothesis [39,40,42,43]. Various forms of cryptospores have also been documented within fragments of the source plants (cryptophytes) that are exceptionally well-preserved in charcoal in Silurian and Lower Devonian sediments [5]. So, we now know that these were minute land plants that resembled the stalk and capsule of the bryophyte sporophyte in general shape and size, but importantly they also differed in some key features, including bifurcation of the stalk (figure 3*m*) and in their method of spore development and dispersal. Cryptospore morphology and dispersal implies a diversity of meiotic cell division in early land plants that is no longer seen in their living descendants [5].

Much remains unknown about early fossil cryptophytes, and because their remains are so fragmentary they have not yet been included in formal phylogenetic analyses. Modern phylogenetic research conclusively demonstrates that land plants are monophyletic [44–46]. The weight of evidence further indicates that bryophytes are paraphyletic to the vascular plants, and that liverworts are sister group to all other land plants (figure 4). However, relations among these basal clades are still uncertain, and various other topologies are plausible, including bryophyte monophyly [46,47]. Phylogenetic analyses group the better known species of *Cooksonia* with the vascular plants rather than the bryophytes (figure 4) [9,11]. These studies also indicate that *Cooksonia*, as originally conceived, is not monophyletic. Species are related in various ways in the stem-groups of the vascular plant and Lycophytina clades. The developing picture is one of cryptophytes representing an extinct pool of diversity from which both modern bryophytes and vascular plants emerged (figure 4) [5].

A second issue revealed by including fossil in phylogenetic analyses is a pattern of representational bias in the geological record greatly favouring the larger and more robust vascular plants over bryophytes and bryophyte-like organisms [11,48]. This bias is less evident in the fossil record of dispersed spores, which tend to have similar dispersal properties and preservational characteristics across groups. A third issue relates to timetrees (i.e. calibrated molecular phylogenies) and estimates for the time of origin and initial diversification of land plants. Timetrees all indicate a lengthier geological history than the fossil evidence, but the degree to which they do so ranges from marginal to substantial [49–51]. The discrepancies among various lines of fossil evidence and timetrees are at least in part related to methodological issues to do with tree calibration. It is probable, however, that these discrepancies also reflect real natural phenomena including significant changes in the nature of the rock record during the Lower Palaeozoic that were operating at a global scale [52].

### (d) The branching sporophyte and nature of gametophyte

One of the key innovations in plant evolution was the capacity for iterative bifurcation of the sporophyte, which laid the foundation for the development of large complex plants with multiple sporangia (polysporangiophytes) and specialized organ systems (e.g. stems, roots, leaves) [53–55]. Vascular plants are polysporangiate whereas bryophytes are not, and *Cooksonia* represents one of the earliest and most rudimentary stages in the evolution of this growth form. Understanding how the polysporangiophyte state first evolved and how it subsequently developed are questions that can be addressed through critical examination of the fossil record and through the methods of evolutionary developmental biology ([56], reviewed in [57,58]). Recently, the evolutionary developmental approach has been greatly enhanced though the establishment of moss model systems [59] and through the sequencing of the genome of *Physcomitrella patens* [60], enabling a comparative genomics approach spanning most of the land plant tree of life [26,61,62].

One emerging area of research focuses on the evolution of indeterminate growth in the spore-bearing part of the plant life cycle. The bryophyte sporophyte is not normally indeterminate, but a capacity for bifurcation in the seta has been known for some time from rare teratological specimens of both liverworts and mosses (figure 3*t*). These are intriguing because they resemble the simple bifurcating sporophyte of Lang's *Cooksonia*. This nascent form of indeterminate growth has been induced in various ways in *P. patens*. Double sporangia were observed in *P. patens* following disruption of homologues of *Arabidopsis thaliana TEL* and *LFY* loci [63,64]. *TEL* encodes a putative RNA-binding protein that plays a crucial role in regulation of post-transcriptional gene expression. *PpLFY* encodes a transcription factor required for the first division of the zygote. Double sporangia were also observed following the application of auxin transport inhibitors [65]. Furthermore, varying the expression of the *PpCLF* gene in the gametophyte results in the apogamous development of bifurcating sporophyte-like structures [66]. *PpCLF* encodes part of the highly conserved Polycomb group (PcG) complex, which is involved in the epigenetic control of gene expression profiles in plants and animals. A key role for PcG in regulating the differentiation of meristematic cells in gametophyte development has also been confirmed through deletion of a *PpFIE* [67]. The disruption of various regulatory mechanisms in *Physcomitrella* is beginning to shed light on the origins of the polysporangiophyte state, as embodied in simple early fossils like *Cooksonia*.

### (e) Non-vascular plants/cryptogamic covers

Today, cryptogamic covers (i.e. communities of cyanobacteria, algae, fungi, lichens, bryophytes) occur on many ground and plant surfaces, where they are responsible for about 7% of the net primary productivity of terrestrial ecosystems [68]. There is a growing body of data indicating that comparable communities dominated the earliest terrestrial ecosystems. Mid-Palaeozoic rock sequences contain many enigmatic fossils that hint at a greater diversity of organisms accompanying and preceding the vascular plant flora. In addition to spores, assemblages frequently contain small fragments of tissue (phytodebris [69]) that resemble the cuticles and complexes of tubes which Lang had assigned to *Nematothallus*. Diverse affinities have been suggested [70]. Recently discovered fossils preserved in charcoal found at sites in the Welsh Borderland are beginning to provide a more complete picture of the *Nematothallus* organism (figure 3*r,s*; [21]). The thallus was stratified with a superficial, usually uniseriate cortex. This confirms Lang's prediction about the underlying structure and the cuticle of *Nematothallus*. The cortex overlaid a palisade zone of parallel tubes with occasional septae and a basal zone of wefts of smaller, randomly interwoven and infrequently branched tubes. This general structure suggests an affinity with lichenized fungi, the tubes being interpreted as fungal hyphae. However, a photobiont has not been observed. A broadly similar type of organization, but lacking the palisade tissue, was observed in two putative lichens from the same locality [71]. Here, there is evidence of a photobiont, with bacterial colonies on the upper surface, and actinobacterial filaments in the medulla. Using material from Lang's fossil collections, Strother [72] calculated that thalloid incrustations of this type accounted for 10–50% coverage of a bedding plane, whereas axial fossils represent approximately 1%. Lichens of nematophyte affinity were probably therefore an important element of early terrestrial vegetation.

Not all coalified thalloid compressions were derived from nematophytes, and most are difficult to interpret because little cellular structure remains. Some have been interpreted as mats of cyanobacteria, whereas others were probably composed of consortia of eukaryotes [73,74]. Stable carbon isotope values (δ^13^C_org_) support a terrestrial origin for much of the fossil-derived carbon in thalloid compression fossils extending back into the Late Ordovician, hinting also at a liverwort affinity [75]. Thus, there is a growing body of evidence for a pre-vascular plant-dominated vegetation, including cyanobacteria, algae, fungi, cryptophytes and lichens on land during the Silurian and Ordovician Periods, comparable with the cryptogamic covers that dominate certain ecosystems today [68].

## 4. Next steps

The genus *Cooksonia* has now been recorded from numerous sites in Europe and the Americas (New York State, Brazil), yet the Welsh Borderland remains the most extensively studied region, yielding important new information on the biology and diversity of these early plants. The charcoal-preserved fossils are unique to this region, and they provide unparalleled insights into tissue systems at the cellular level. The research tool of choice has been the scanning electron microscope, which enables the analysis of cell structure but is limited in its capacity to build a complete three-dimensional model of the organism. X-ray synchrotron microtomography (SRXTM) provides a new and very promising alternative. This non-destructive technique has been applied to imaging the organs and tissue systems in fossil plants, yielding full three-dimensional models [76]. SRXTM has been used to image minute charcoalified seeds and flowers from the Cretaceous Period [77,78], which resemble *Cooksonia* in their preservation, size fraction and in their rarity. SRXTM, therefore, has the potential to yield complete reconstructions of the soft tissue systems of *Cooksonia,* shedding further light on the functional morphology of the earliest land plant.

Furthering our understanding of the cryptophytes (i.e. plants that produced the cryptospores that characterize Ordovician through Devonian rocks) and associated organisms requires both the identification of appropriate rock sequences in the field and the application of new methods. New data from Gondwana would be of great interest as there are very few early records from this continent [79]. The extraction and description of mesofossils (millimetre scale fossils that fall into the size range between spores and macroscopic compressions) using acid maceration techniques and *in situ* imaging will form an important part of the approach [5].

Evolving the capacity to maintain an internally hydrated environment under water stress (homoiohydry) was of fundamental importance in the colonization of the land. The evolution of homoiohydry involved the acquisition of a suite of features including a cutinized epidermis, stomata, gas-filled intercellular spaces (air-spaces) and vascular tissue [80]. Modern bryophytes possess some of these features, and their functions are now known to differ in some important respects from the better studied vascular plants [30]. Also, there are important differences in the chemistry of secondary compounds that X-ray spectroscopy may well distinguish. Unravelling the evolution of homoiohydry requires a better understanding in bryophytes of the nature and roles of lignin-related polyphenolics, cuticle chemistry and structure, and the function of the stomatal apparatus in relation to their distributions and the absence of intercellular spaces and water-conducting tissues.

The establishment of a moss model system and the sequencing of the genome of *P. patens* now enable a comparative genomics approach spanning most of the land plant tree of life [59,60]. The disruption of various regulatory mechanisms is beginning to shed light on the origins of the polysporangiophyte state [63–66], as embodied in simple early fossils like *Cooksonia*. Likewise, genetic and transcriptomic approaches promise to reveal mechanisms regulating the land plant life cycle [81]. The extension of these approaches to other key living groups, including hornworts, liverworts and charophycean algae is a necessary step in understanding the evolution of basic organs and tissue systems in plants. Also, the emerging molecular developmental models need to account for the morphology and life-cycle variants of extinct plants [82].

The phylogenetic tree of plants is the framework that underpins evolutionary interpretations, but several important relationships are still poorly resolved. The sister group of land plants within living charophycean algae is unclear (Charales/Zygnematales) [46,83,84]. This has implications for understanding the likely suite of features that characterized the common ancestor of land plants. Similarly, the relationships among the major clades of bryophytes and the vascular plants are still problematic, with low support for any one topology [46,47]. Whether bryophytes are monophyletic or paraphyletic to the vascular plants has important implications for our understanding of the evolution of the land plant life cycle and the evolutionary implications of early fossils.

Lang's discoveries in the Welsh Borderland brought to light hitherto unknown very small fossil plants and other enigmatic fossilized remains that opened a window on to the nature of early terrestrial ecosystems. Some of the novel organisms he described proved to be related to plants, whereas the affinities of others are still uncertain, but they are likely to be quite diverse within the eukaryotic tree of life. In modern terms, Lang's discoveries showed that early terrestrial ecosystems resembled modern soil crust or epilithic microbial communities that were dominated by cryptogamic plants. The plants were small, could not be classified comfortably in established families, and lacked many of the features associated with modern species. The iconic *Cooksonia* in particular has served as a starting point in thinking about the evolution of basic organs and tissue systems (e.g. vascular system, stem, roots, leaves). Subsequent research has clarified and extended this work. Evidence from minute fossils provides the main source of information on the composition and evolution of early terrestrial ecosystems, and new techniques and analytical approaches are helping to solve questions concerning the biology and affinities of these organisms. Molecular phylogenetics now provides a robust framework within which to appraise this evidence, and molecular developmental biology a new approach to understanding and explaining the evolution of plant form. The broader impact of early land-based life on key Earth Systems (e.g. geochemical carbon cycle) is a developing area of research [85–87].

Lang concluded his magnum opus with the statement that his ‘present study and survey of the Downtonian flora will, it may be hoped serve as a basis of such further work′—and so it turned out to be, with much wider ramifications into areas undreamed of by this superlative botanist.

## Author profiles

**Dianne Edwards** is a distinguished research professor in palaeobotany at Cardiff University, where she has researched early land plant fossils for the past 45 years. Her studies have involved collaborations and fieldwork with palaeobiologists in Australia, Argentina, Bolivia, Venezuela and China, but she has concentrated mainly on Silurian and Devonian rocks of South Wales and the Welsh Borderland, resulting in a total of more than 165 publications. Distinctions include medals from the Linnean and Geological Societies and the Palaeontological Association, honorary degrees from Dublin and Uppsala, and election to the Royal Societies of London and Edinburgh, the Learned Society of Wales and Academia Europaea. She has been president of the Linnean Society and Palaeontological Association.

**Paul Kenrick** is a research scientist at the Natural History Museum, London. His interest in the evolution of land plants was sparked by an early encounter as a student with the tiny fossil plant *Cooksonia*. His career has been based in museums, including the Swedish Museum of Natural History, Stockholm, and the Field Museum, Chicago, where he integrated fossil evidence on early plants with the emerging phylogenetic picture based on living species. From 2008 to 2011, he was visiting professor in Plant Sciences at the University of Oxford. Kenrick has received several awards including the Henry Allan Gleason Award of the New York Botanical Garden and the Bicentennial Medal of the Linnean Society of London. He has authored two books and over 60 peer-reviewed publications.
